# Mesoporous silica nanocarriers encapsulated antimalarials with high therapeutic performance

**DOI:** 10.1038/s41598-018-21351-8

**Published:** 2018-02-15

**Authors:** Saliu Alao Amolegbe, Yui Hirano, Joseph Oluwatope Adebayo, Olusegun George Ademowo, Elizabeth Abidemi Balogun, Joshua Ayoola Obaleye, Antoniana Ursine Krettli, Chengzhong Yu, Shinya Hayami

**Affiliations:** 1 Department of Chemistry, College of Physical Sciences, Federal University of Agriculture, Abeokuta (FUNAAB) PMB, Abeokuta, 2240 Nigeria; 2 Department of Chemistry, Graduate School of Science and Technology, Kumamoto University, 2-39-1 Kurokami, Chuo-ku, Kumamoto, 860-8555 Japan; 3 Department of Biochemistry, Faculty of Life Sciences, University of Ilorin, PMB 1515 Ilorin, Kwara State Nigeria; 4 Institute for Advanced Medical Research and Training (IAMRAT) College of Medicine University College Hospital, University of Ibadan, Ibadan, Nigeria; 5 Department of Chemistry, Faculty of Physical Sciences, University of Ilorin, PMB 1515 Ilorin, Kwara State Nigeria; 6 Laboratorio de Malaria, Centro de Pesquisas Rene Rachou, FIOCRUZ, Belo Horizonte, 30130-100 MG Brazil; 7 Australian Institute for Bioengineering and Nanotechnology, The University of Queensland, Queensland, QLD 4072 Australia; 8 Institute of Pulsed Power Science (IPPS), Kumamoto University, 2-39-1 Kurokami, Chuo-ku, Kumamoto, 860-8555 Japan

## Abstract

The use of nanocarriers in drug delivery is a breakeven research and has received a clarion call in biomedicine globally. Herein, two newly nano-biomaterials: MCM-41 encapsulated quinine (MCM-41 ⊃ QN) (**1**) and 3-phenylpropyl silane functionalized MCM-41 loaded QN (pMCM-41 ⊃ QN) (**2**) were synthesized and well characterized. **1** and **2** along with our two already reported nano-antimalarial drugs (MCM-41 ⊃ ATS) (**3**) and 3-aminopropyl silane functionalized MCM-41 contained ATS (aMCM-41 ⊃ ATS) (**4**) were screened *in vitro* for their activity against *P. falciparium* W2 strain, cytotoxicity against BGM cells and *in vivo* for their activity against *Plasmodium bergheiNK65*. **1** has the highest antimalarial activity *in vivo* against *P. berghei* NK65, (ED_50_: < 0.0625 mg/kg body weight) and higher mean survival time compared to the other nano biomaterials or unencapsulated drugs at doses higher than 0.0625 mg/kg body weight. This encapsulation strategy of MCM-41 ⊃ QN (**1**) stands very useful and effective in delivering the drug to the target cells compared to other delivery systems and therefore, this encapsulated drug may be considered for rational drug design.

## Introduction

**Figure 1:**
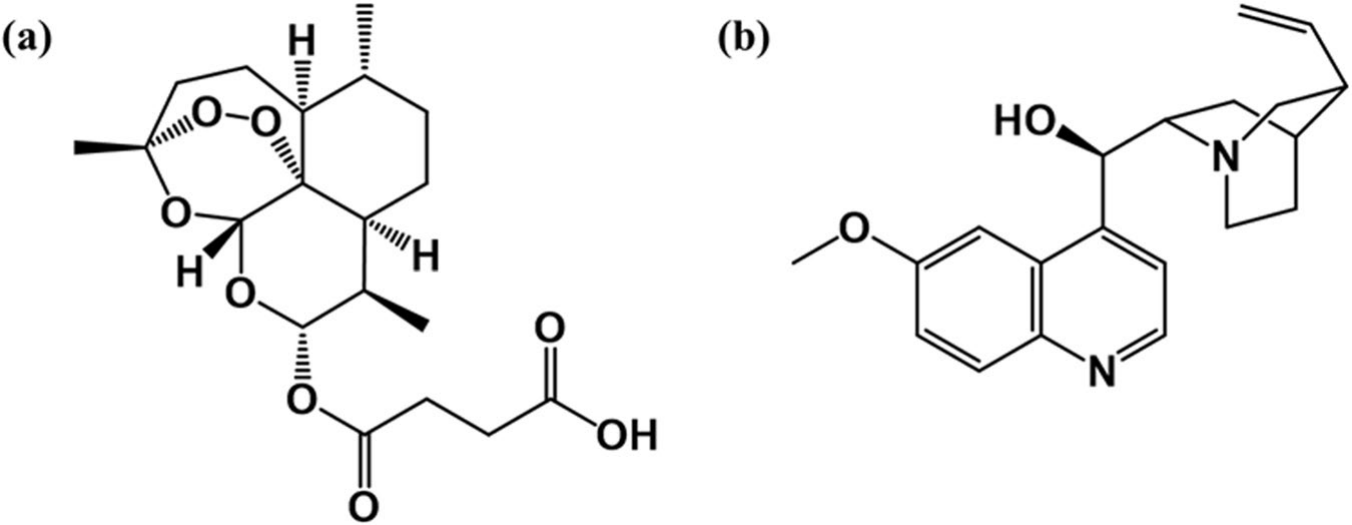
Chemical structures of (**a**) ATS and (**b**) QN.

Delivery of antimalarial drugs to their target site through innovative drug delivery system with the goal of overcoming emerging global resistant parasites has been one of the commitments of World Health Organization^1^. Unfortunately, the potency of these drugs (either as single or combined therapy) towards complete clearance of *Plasmodium* species is still far below the satisfactory level due to drug resistance developed by these parasite and this may contribute to high estimated deaths which was 438,000 in 2015^1^. This aforementioned fact, is attributed to the hydrophobicity/poor aqueous solubility of drugs in water, instability and decomposition among other factors which are responsible for their reduced bioavailability, and/or the survival of the parasite^2–4^. The high incidence of malaria, being the most dreaded human parasitic disease in the tropic and sub-tropic countries and drug-resistance cum toxicity turned the disease into a problem of major health importance^5,6^. Some of these drugs have short half lives in the blood, e.g. artesunate (ATS), thereby leading to the problem of recrudescence while some of them exert some adverse effects at their therapeutic doses^7^, e.g. quinine (QN) Fig. 1. These challenges can be overcome by increasing the half-lives of the drugs in the blood, reducing the doses which normally exert the unwanted adverse effects, though increasing the amount of the drugs that gets to the target cell^8,9^.

**Figure 2:**
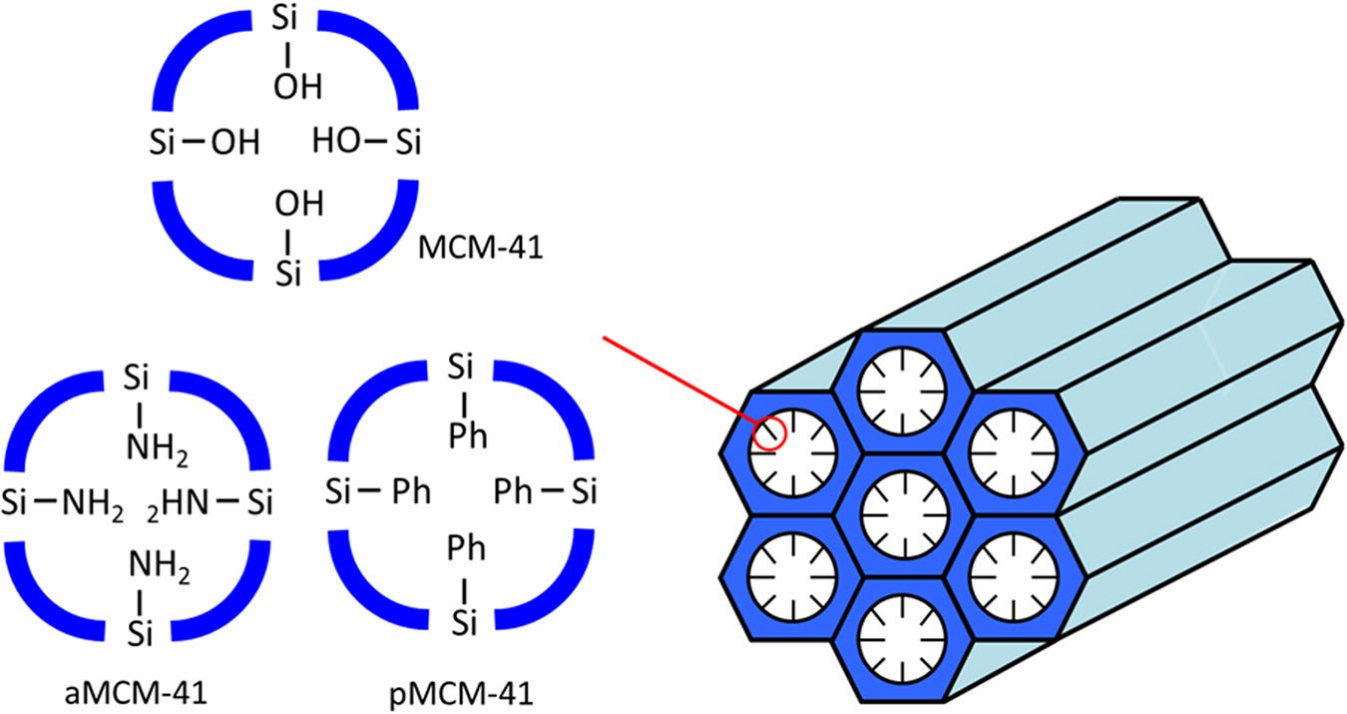
Scheme of surface phenyl organo-functionalization of MCM-41 encapsulated quinine.

Nanomedicineis a new drug design rational which is useful, versatile and smarter small sized drug delivery platform compared to other long existing therapeutics notably phytomedicine, metal based drugs and so on^10–14^. Development of very sensitive and novel means of delivering antimalarial drugs through nanovehicle drug delivery system (DDS) stands clear as a promising option in chemotherapy. This was inspired by medicinal inorganic professionals for arresting the impotency of many antimalarial drugs in recent time^11^. Interestingly, nanoveschiles lipid compliance as a rationale drug design was recently used to derive the local and global organization of a multi-mega Dalton peptide-based nanaocarrier. It renders a high molecular detail and at close-to physiological conditions^15^. Among nanocarriers for the release of biomolecules which had been researched upon include liposomes, dendrimers polymers, clay, zeolites to mention a few^16,17^. However, the utilization of mesoporous silica nanoparticles (MSNs) as a strategy in drug design application came as a better alternative nanocarrier due to its inherent noble properties of large surface area, tuneable pore size or volume, high thermal property, nontoxicity, biocompatibility which enables it cargo favourably, large drug proportions to the target cells in a controlled kinetic release^18,19^. Beck *et al*.^20^ were the first to introduce MSN popularly referred to as MCM-41 in 1992. Besides, surface functionalization of the silanol groups towards robust inorganic-organic frame work can also enhance encapsulation of drugs, our case study of phenyl surface modification of silanol walls for quinine encapsulation^21,22^ (Fig. 2). Construction of nano-vector materials, capable of encapsulating antimalarial drugs and delivering them to *Plasmodium*-infected red blood cells (pRBC) with high specificity and efficacy and at an affordable cost to the rural man in sub-Sahara Africa is of particular interest. Urban *et al*.^23^ reported the use of poly (amidoamines) (PAAs) drug conjugates for the delivery of chloroquine (CQ) and primaquine (PQ) to *P. falciparium 3D7 in vitro* with IC_50_ values of 14.6 nM and 2.5 µM respectively when the most effective PAA (having an intrinsic antiplasmodial activity, IC_50_ of 13.7 µM was used and to *P. yoelii*17XL *in vivo* with 96.5% reduction in parasitemia at the least dose when the most effective PAA was also used. Movellanand co-workers^24^ constructed nano dendritic polymer drug conjugates for CQ and PQ carriage to *Plasmodium*-infected red blood cell (pRBC) with a clear improvement in the vitro IC_50_ for CQ and PQ which were 4.0 nM and 1.1 µM respectively.

Despite the recent success story of artemisinin derivatives as effective antimalarial drugs, the emergence of resistance and the quest to arrest lukewarm status of QN, the first line antimalarial drug for complicated malaria, are still challenges in chemotherapy that requiresdrastic improvement^25,26^. Nevertheless, ATS (a derivative of artemisinin) and QN (Fig. 1), either in a single or combined therapy through oral route, are susceptible to the development of resistance by *Plasmodium* strains and the lipophilic property of the latter may be a contributing factor.

Various types of polymeric vessels, liposomes, e.g. neutral multi-lamellar liposomes with cholesterol modifications as nanocarriers have been used as support to facilitate the release of many antimalarial drugs to the infected red blood cells^27^ Particularly, the release of ATS from the neutral liposomes had been influenced by the lipid content in the liposomal bilayer, liposome dimension and drug release kinetics^28^.

In the search for improvement of the potency of antimalarial drugs, we set the hypothesis on novel nanocarrier MSNs envelopes/cargoesas good drug delivery system for QN and ATS, bearing in mind theirunique pore dimensions with high adsorptivities. Meanwhile, our previous research work on MSNs captured synthesis of MCM-41 and surface organically modified silica hybrids as vehicle for an antimalarial drug, ATS, with very high loading strength and satisfactory slow pharmokinetic release^29^. To the best of our knowledge as evidenced from literatures search, there has been no report on the use of MSNs for ATS and QN delivery system. It is therefore necessary to evaluate such antimalarial loaded nanoparticles for their antimalarial activities. This study was therefore carried out to synthesize two new MSNs loaded QN and evaluate their delivery performance alongside with another two known nano-silica encapsulated artesunate drug (ATS) with the aim of effectively delivering the antimalarial drugs to Plasmodium-infected red blood cells and subsequent clearance of the parasites.

## Results

### Nano-silica encapsulated quinine drugs (*NSEQD*)

A sol-gel synthetic method previously reported for the parent silica MCM-41 and 3-aminopropyl silane functionalized MCM-41 (aMCM-41) was followed with slight modification to carry out the syntheses of the MCM-41 and 3-phenylpropyl silane functionalized silica (pMCM-41)^30^ (Fig. 2, see the Supporting Information). QN loading and release experiment were carried out as well for (MCM-41 ⊃ QN) **1** and (pMCM-41 ⊃ QN) **2** were similar to previous reports on (MCM-41 ⊃ ATS) **3** and (aMCM-41 ⊃ ATS) **4** nano drugs. The drug free MSNs prepared exhibited properties similar to our previous reported silica materials^29^. However, the loading of QN inside the pore of MSNs depicts new physicochemical properties for **1** and **2**. The powder X-ray diffraction (PXRD) measurements were performed for investigating the well-ordered structure of MCM-41, **1** and **2**. Three diffraction peaks of the MCM-41 nanosilica were observed at 2*θ* angle of 2.63°, 4.34° and 5.03° which can be indexed to (100), (110) and (200) planes associated with a *p6mm*hexagonal symmetry (Fig. S1), similar to report in literatures^31^. Meanwhile, free QN showed strong peaks at 2*θ* angle of 5.15° and 15.7° which cannot be observed in the diffraction pattern of the loaded drugs (**1** and **2**) due to QN’s impregnation in the MSNs with amorphous state. The Fourier transform infrared (FT-IR) in the range of 4000–400 cm^−1^ was employed to check the expected functional groups or bonds in the silica materials and QN adsorbed silica interactions. In the as-synthesized MCM-41, C-H stretching vibrational frequency was due to surfactant attachment absorbed around 2916–2855 cm^−1^ but for pMCM-41, peak at 2923 cm^−1^ showed the propyl substituent. In the template-free MCM-41, 1080 cm^−1^ and 957 cm^−1^ depict the characteristic stretching and bending vibrational frequency of -Si-O-Si- fragment while broad peak at 3456 cm^−1^ is attributed to the silanol group (Fig. S2). Similar peaks of the parent MSNs were found in the spectra of the nanodrugs (**1** and **2**) but with reduced intensities due to QN interaction within the silica moieties during loading. Strong and sharp absorption band around 2932 cm^−1^ and finger print around 1400 cm^−1^ are the C-H alkane bond in the spectrum of QN, which disappeared in the spectrum of the silica loaded QN due to encapsulation by silica molecules (Fig. S3)^32^.The Differential Scanning Calorimetry (DSC) measurement depicts the structural phase transition of the free QN at 400 K (Fig. S4). The sharp melting enthalpy did not appear in the silica encapsulated QN, which agrees, with the amorphous state of the drug earlier revealed from PXRD already stated and Fourier Transform Infrared Spectroscopy (FTIR) result to be presented later. The MSNs free drug had no absorption within the endothermic scanning range (25–460 K) confirming its thermal stability. The thermogravimetric analysis (TGA) data expresses the weight loss of QN (Fig. S6). Temperature around 75 °C is responsible for the weight loss due to solvent effect (ethanol) used for loading. When the temperature is above 250 °C, more than 70% of QN had been decomposed in the free QN system. The MSN-QN remained undecomposed even above 500 °C as the drug was totally shielded from decomposition due to the silica envelope^33^.

**Figure 3:**
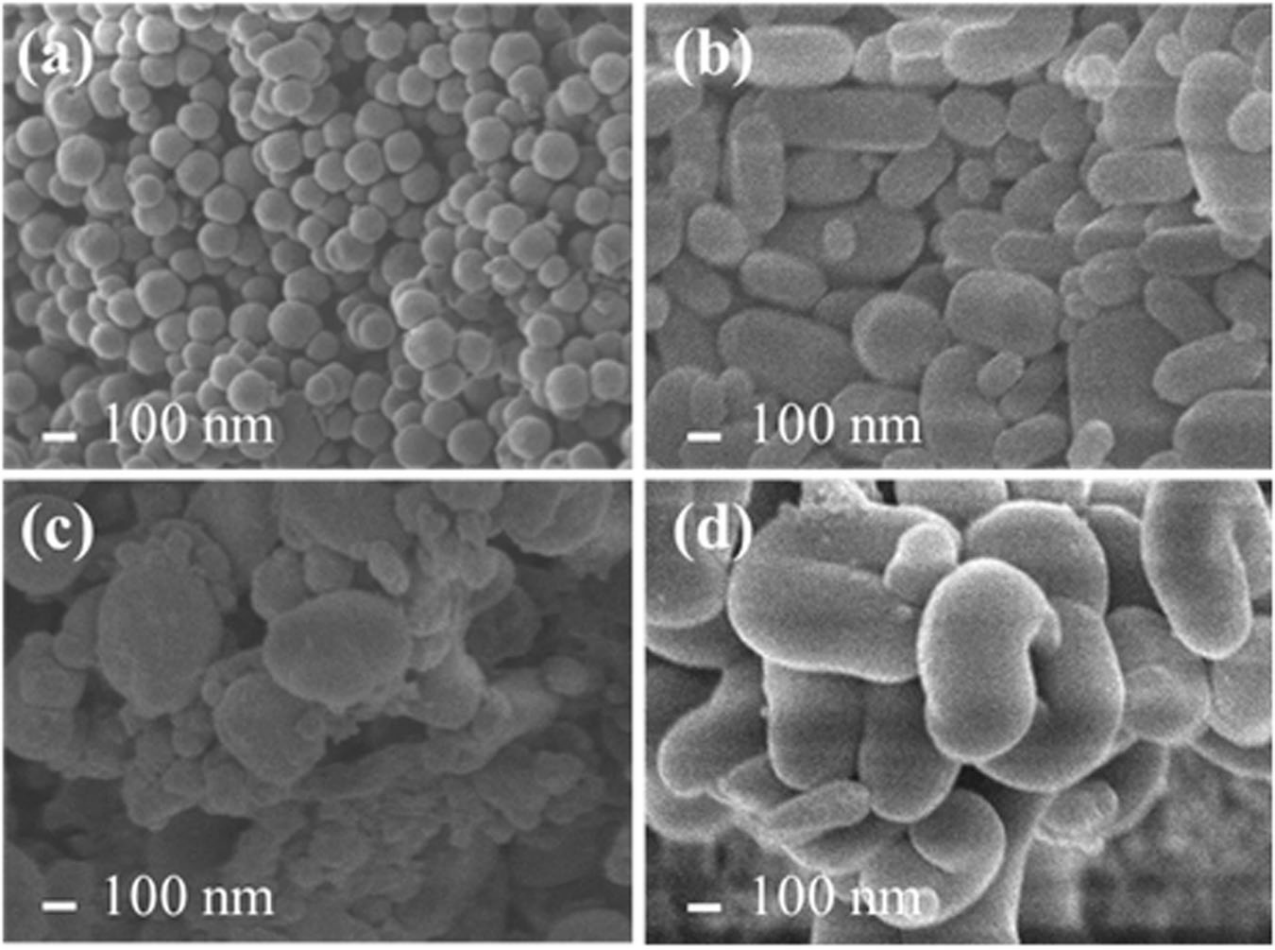
SEM images of (**a**) MCM-41, (**b**) **1**, (**c**) pMCM-41 and (**d**) **2**.

The structural image and surface morphology of the MSNs and nanosilica QN loaded were characterized using Transmission Electron Microscope (TEM) and Scanning Electron Microscope (SEM) (Fig. S5 and Fig. 3). The respective long hexagonal and complete u-tube surface micrograph of **1** and **2** respectively from the SEM micrographs are different from the monodispersed spherical shape of MCM-41. This lends credence to the fact that QN had been encapsulated, which is capable of reordering the silica spherical shape through the adsorption and chemical interaction within the silanol surface and hydrogen bonded aromatic silylating functional group. The pMCM-41 showed some little curved hexagonal shape as early reported but with presence of significant bulky oval silica shape due to phenyl intervention.

The silica interior pore properties were measured with N_2_ adsorption/desorption isotherms techniques (Fig. S6 and Table 1). The specific surface area using Brunauer-Emmett-Teller (BET), the pore size by Barrett-Joyner-Halenda (BJH) and the total pore volumes of MCM-41 were 1.498 × 10^3^ m^2^ g^−1^, 2.18 nm, and 0.53 cm^3^ g^−1^, respectively. There was a noticeable decrease in the pore properties of **1** (453.33 m^2^ g^−1^, 2.06 nm, and 0.23 cm^3^ g^−1^) attributed to the partially occupied surface silanol group by QN encroachment^24^. 3-PPS (262.06 m^2^ g^−1^, 2.38 nm, and 0.16 cm^3^ g^−1^), similar trend was observed for **2** (256.76 m^2^ g^−1^, 2.33 nm, and 0.15 cm^3^ g^−1^) which is due to the drug encapsulation but with greater payload of QN attributed to the organic-organic interface^34^. The adsorption of QN to the silica walls was successfully done by soaking silica materials in the ethanol solution of QN with continuous stirring for 3 days. Both neutral pH 7.4 and adjusted pH 2.0 of the 0.5% sodium lauryl sulphate (SLS) buffer with 0.1 M hydrochloric acid (HCl) were used to investigate the release of QN from nanosilica loaded drug.

**Table 1:** N_2_ sorption data and loading property of MSNs.

Materials	S_BET_ (m^2^ g^−1^)	W_BJH_ (nm)	V_p_ (cm^3^ g^−1^)	DLC (%)	EE (%)	Adsorbed drug (mg)
MCM-41	1498.31	2.18	0.54	—	—	—
pMCM-41	262.06	2.38	0.16	—	—	—
**1**	453.33	2.07	0.23	3.96	15.90	1.59
**2**	256.76	2.33	0.15	8.72	36.10	3.61
QN	—	—	—	—	—	—

DLC = drug loading capacity, EE = entrapment efficiency.

**Figure 4:**
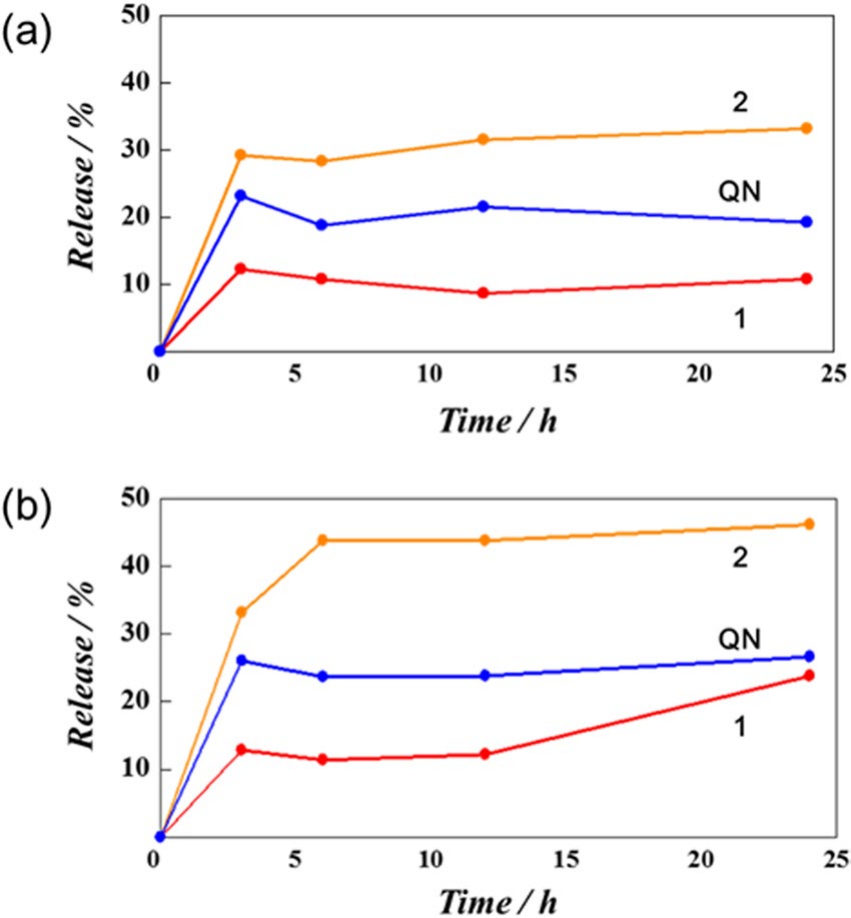
Dissolution profiles of QN, **1** and **2** in (4: 1) system, 0.5% SLS buffer at 37 °C with the (**a**) pH = 7.4 and (**b**) pH = 2.0.

The amount in percentage of QN adsorbed onto the silica materials are expressed in terms of drug loading capacity (DLC) and entrapment efficiency (EE) which are calculated using quantitative UV-Vis spectrophotometer at QN absorption characteristic wavelength of 332 nm as summarized in the Table 1. The BET surface area and structural assembly of MSNs affected the loading affinity in line with many literature viewpoints^35^. It was observed that functionalization of silanol surface with organo phenylpropyl silylating agent,is a good strategy towards increasing QN adsorption (3.61 mg, 8.72%) proven to be a good strategy towards increasing drug adsorption unlike the free MCM-41 (1.59 mg, 3.96%) despite its higher S_BET_ of the latter. From the kinetic release, it was observed that the result of QN release in the buffer dialysis membrane medium depend on pore size of the MSNs and pH medium^36^ (Fig. 4) The acidic system experienced a better kinetic release than the neutral environment for QN across the test times (0–24 h). Free QN had its appreciable release up to 23.15% and 26.05% in neutral and acidic media respectively within 3 h. It suffices to attribute this optimal bursting behavior of QN within a relative short time to be due to its release outside the encapsulant (i.e. MSNs) which is capable of causing toxicity to the normal cells *in vivo*. As expected, **2** with largest pore size was followed with a relative better controlled release of 33.18% release for 3 h in the neutral medium and 46.15% release after 24 h in acidic medium. We observed a very gradual slow release pattern for **1**, with 12.9% release after 3 h in the neutral medium and 23.8% in 24 h from the acidic environment attributed to its small pore size and strong chemical interaction between silanol group and QN^37^.

### Antiplasmodial activity and cytotocity of the nanodrugs

The anti-plasmodial activity and cytotoxicity for the nanodrugs: MCM-41 ⊃ QN (**1**) pMCM-41 ⊃ QN (**2**), MCM-41 ⊃ ATS (**3**) and aMCM-41 ⊃ ATS (**4**) were evaluated. Apart from MCM-41 encapsulated ATS, the other three nanoparticle encapsulated antimalarial drugs were active against *P. falciparum W2* strain and were not toxic against Buffalo Green Monkey Kidney (BGM) cells (Table 2).

**Table 2:** Antiplasmodial activities and cytotoxicities of nanoparticle encapsulated antimalarials (*in vitro*).

Nanodrugs/Drugs	Activity against P. falciparum (clone W2) -IC_50_ (*μ*g/mL)			Cytotoxicity against BGM Cells-MLD_50_ (*μ*g/mL)			Selective Index (SI)	Remarks
	Exp. 1	Exp. 2	Mean ± SD	Exp. 1	Exp. 2	Mean ± SD	MLD_50_/IC_50_	—
1	1.80	1.70	1.75 ± 0.07 (0.11 Qn)	>1000	>1000	>1000 ± 0	571	Active
2	1.70	1.50	1.60 ± 0.14 (0.10 Qn)	>1000	>1000	>1000 ± 0	625	Active
3	>50	>50	>50 ± 0 (>8.33 As)	>1000	>1000	>1000 ± 0	—	Inactive
4	10	8.20	9.10 ± 1.00 (1.52 As)	>1000	>1000	>1000 ± 0	110	Active
QN	0.17	0.58	0.38 ± 0.30	140	166	153 ± 18	403	Active
ATS	0.15	0.13	0.14 ± 0.01	198	112	155 ± 61	1107	Active

Each experiment (Exp) was conducted in triplicate. The values in the brackets are for the drugs only without the nanoparticles.

### Antimalarial activity of nano-silica encapsulated antimalarial drugs

The results of the 4-day suppressive test using mice also revealed that **1** was the most active nanodrug (ED_50_: < 0.0625 mg/kg) against *P. berghei* NK65 on day 4 post-inoculation (Tables 3 and 7), exhibiting higher chemosuppression than quinine at a dose two hundred and forty times less than that of QN and also increased the mean survival time (MST) compared to the untreated control. Moreover, the MST exhibited by **1** was comparable to that exhibited by QN in the infected mice. **3** was the second most active against *P. berghei NK65* (ED_50_: 0.113 mg/kg) and also increased the MST of the infected mice compared to the untreated control (Tables 5 and 7). However, it exhibited lower MST in infected mice compared to ATS. The other two nanodrugs (**2** and **4**) were also active; causing more than 50% inhibition of parasite growth on day 4 post-inoculation at higher doses (Tables 4 and 6). However, as others, the nanodrugs caused the *P. berghei NK65*-infected mice to have lower MST than those administered standard drugs, though increasing MST of the infected mice compared to the untreated control.

**Table 3:** Antimalarial activity of MCM-41 encapsulated QN (**1**) in *P. berghei NK65*-infected mice.

Dose (mg/kg bw)	Parasitemia (% Reduction)			Mean Survival Time (Days)
	Day 4 post-inoculation	Day 6 post-inoculation	Day 8 post-inoculation	
Infected Untreated	2.81	3.51	9.60	7.75
**QN1** (15)	0.90(68.14)	0.97(65.48)	2.30(76.06)	20.50
0.0625	0.25(91.10)	0.38(89.47)	0.85(90.71)	16.67
0.125	0.02(99.18)	0.07(98.03)	0.12(99.50)	23.33
0.25	0.40(85.65)	0.69(80.83)	0.89(90.25)	26.67
0.50	0.14(95.12)	0.54(84.93)	0.52(94.33)	21.67
1.00	0.48(82.77)	0.42(88.28)	0.31(96.61)	18.00

Values are expressed as means of 5 replicates, bw: body weight, **QN 1** (15) : MCM-41 ⊃ QN, 15 mg/kg body weight of QN.

**Table 4:** Antimalarial activity of pMCM-41 encapsulated QN (**2**) in *P. berghei NK65*-infected mice.

Dose (mg/kg bw)	Parasitemia (% Reduction)			Mean Survival Time (Days)
	Day 4 post-inoculation	Day 6 post-inoculation	Day 8 post-inoculation	
Infected Untreated	2.81	3.61	9.60	7.75
**QN2** (15)	0.90(67.97)	0.98(73.13)	2.30(76.04)	20.50
0.0625	2.59(7.83)	1.14(68.42)	1.63(83.02)	16.50
0.125	2.58(8.19)	0.55(84.76)	2.86(70.21)	16.50
0.25	1.59(43.42)	0.69(80.89)	1.43(85.10)	20.50
0.50	0.87(69.04)	0.73(79.78)	12.13(26.35)	13.25
1.00	0.51(81.85)	0.69(80.89)	1.24(87.08)	17.50

Values are expressed as means of 5 replicates, bw: body weight, **QN 2**(15) : pMCM-41 ⊃ QN, 15 mg/kg body weight of QN.

**Table 5:** Antimalarial activity of MCM-41 encapsulated ATS (**3**) in *P. berghei NK65*-infected mice.

Dose (mg/kg bw)	Parasitemia (% Reduction)			Mean Survival Time (Days)
	Day 4 post-inoculation	Day 6 post-inoculation	Day 8 post-inoculation	
Infected Untreated	2.81	3.61	9.60	7.75
**ATS3** (5)	0.45(83.99)	0.97(73.13)	2.30(74.86)	23.47
0.0625	1.55(44.84)	0.82(77.28)	0.28(96.94)	13.01
0.125	1.88(33.10)	1.40(61.22)	1.55(83.06)	11.01
0.25	0.12(95.73)	1.61(55.29)	0.09(98.97)	16.30
0.50	0.30(89.32)	1.70(55.40)	0.09(98.97)	12.33
1.00	0.59(79.00)	0.27(92.52)	0(100)	11.50

Values are expressed as means of 5 replicates, bw: body weight, **ATS 3** (5) : MCM-41 ⊃ ATS, 5 mg/kg body weight of ATS.

**Table 6:** Antimalarial activity of aMCM-41 encapsulated ATS (**4**) in *P. berghei NK 65*-infected mice.

**Dose (mg/kg bw)**	**Parasitemia (% Reduction)**			**Mean Survival Time (Days)**
	**Day 4 post-inoculation**	**Day 6 post-inoculation**	**Day 8 post-inoculation**	
Infected Untreated	2.81	3.61	9.60	7.75
**ATS4** (5)	0.45(83.99)	0.68(81.16)	2.04(78.75)	13.50
0.0625	2.73(2.85)	1.16(67.87)	4.85(49.48)	15.75
0.125	0.09(96.80)	4.99(38.23)	5.43(43.44)	13.50
0.25	0.86(69.40)	2.24(37.95)	7.65(20.31)	18.25
0.50	0.81(71.17)	1.76(51.25)	8.54(11.04)	16.50
1.00	0.00(100)	1.47(59.28)	0.56(94.17)	9.00

Values are means of 5 replicates. bw: body weight, **ATS 4** (5) : aMCM-41 ⊃ ATS, 5 mg/kg body weight of ATS.

**Table 7:** ED_50_ of Nanoparticle encapsulated antimalarials.

Nanodrug	ED_50_ (mg/Kg body weight)		
	Day 4 post-inoculation	Day 6 post-inoculation	Day 8 post-inoculation
1	<0.0625	<0.0625	<0.0625
2	0.327	ND	0.218
3	0.113	0.458	<0.0625
4	0.157	0.454	0.157

ED_50_: Effective dose causing 50% chemosuppression.

The results identified **1** and **3** as the most active nanodrugs, though the latter was inactive *in vitro*. This suggests that MCM-41 was the most effective drug delivery system among the drug delivery systems examined. It also suggests that the nanoparticle used for the synthesis of **3** might have undergone some modifications *in vivo* which enhanced the release of the drug from the nanoparticle thereby making the nanodrug to become active. **1** exhibited an ED_50_ of <0.0625 mg/kg body weight on days 4, 6 and 8 post-inoculation, which was lower than **2** in the respective days. The results suggest that MCM-41 was able to maintain a steady release of the drug over a long period of time, thereby increasing the half-life of the drug in the blood. This is also evident from the *in vitro* dissolution experiment carried out. In like manner, **3** exhibited lower ED_50_ than aMCM-41 encapsulated ATS on days 4 and 8 post-inoculation, still buttressing the fact that MCM-41 is effective at steady release of its drug compared to other nanoparticles. This also suggests that the frame work of MCM-41 was effective in allowing the nanodrug to get adsorbed to the infected red blood cells.

## Discussion

We have synthesized well-ordered drug carriers MCM-41 and 3-phenylpropyl silane organo functionalized silica (pMCM-41), as new biomaterials MSNs pay load for QN. All the physical characterizations of the biomaterials reveal the QN encroachment in the silica mesopores. The kinetic release study depicts a better controlled release of **1** at acidic condition over free QN which burst into the buffer medium under 3 h. These two newly synthesized nano-vehicles of QN and already two known ATS containing silica nanomaterials (MCM-41 ⊃ ATS and aMCM-41 ⊃ ATS) were evaluated for their effectiveness in the release of antimalarial drugs to the infected red blood cells both *in vivo* and *in vitro*. MCM-41 encapsulated QN (**1**) with a controlled release was the most active of all the four nanodrugs evaluated, causing higher inhibition of parasite growth than the parent drug and exhibiting a mean survival time favourably compares with that of parent drug. The results therefore, suggest that **1** is more effective drug delivery system compared to other nanoparticles used in this study. Thus, its application as a drug delivery system for the antimalarials makes it a suitable candidate for the next generation active nano drugs malariotherapy products^10,11^.

Moreover, MCM-41 enhanced a dose of 0.0625 mg/kg body weight of QN (which is 240-fold less than 15 mg/kg body weight of the unencapsulated QN used in this study) to cause a higher inhibition of parasite growth compared to the unencapsulated drug. In the same vein, MCM-41 enhanced a dose of 0.25 mg/kg body weight of ATS (which is 20-fold less than the 5 mg/kg body weight of the unencapsulated ATS used in this study) and interestingly still to cause a higher inhibition of parasite growth compared to the parent drug. This result, however, corroborates the high intracellular uptake property of MSNs^18–20^. The magnitude of the reduction in the amount of drug needed to cause higher inhibition in parasite growth than the unencapsulated drugs was higher in MCM-41, suggesting that MCM-41 further enhanced its effectiveness in drug delivery.

The nanodrugs increased the mean survival time of the infected mice compared to that of the untreated control. However, the reduced mean survival time observed in mice treated with various mesoporous silica nanoparticles loaded with antimalarials, except MCM-41 encapsulated quinine, compared to those treated with parent drugs suggests some level of toxicity. The nanodrugs were not toxic against Buffalo Green Monkey Kidney cell line *in vitro* (MLD_50_: > 1000 in Table 2). Thus, the cause of the reduced MST observed *in vivo* is a subject for further studies. The results corroborate previous reports that unfunctionalized nanoparticles (e.g MCM-41) are well tolerated^13^. MCM-41 encapsulated QN had comparable mean survival time to that of QN, even exhibiting higher mean survival time than quinine at some doses which were much less than that of unencapsulated quinine. This suggests that it is relatively safe as a drug delivery system compared to the other three mesoporous silica nanoparticles (MSNs). The results of this study suggest that, of the three mesoporous silica nanoparticles used as drug delivery systems, MCM-41 was the most effective and its drug conjugate may be considered for rational drug design.

## Methods

### Chemicals and Life animals for *in vivo*

All the chemicals were of spectroscopic grade and were used without any further purification: Tetraethyl orthosilicate (TEOS), cetyl trimethyl ammonium bromide (CTAB), and 3-phenylpropyltrichlorosilane (3-PPS) were purchased from Sigma Aldrich, sodium lauryl sulphate (SLS) from Wako, dialysis membrane. Quinine drug (QN) was purchased from TCI. Deionized water, AR grade was used for all the preparation and purification. Giemsa stain was obtained from Anosantec Laboratories, UK. Methanol was obtained from Eagle Scientific Ltd., Nottingham. Immersion oil was obtained from Panzonar Laboratory Supplies, Button road, Canada. RPMI 1640 medium, sodiumbicarbonate, L-glutamine, D-sorbitol, and HEPES were obtained from Sigma (St. Louis, MO, USA). All other reagents were of analytical grade and were prepared in all glass distilled water.

A Chloroquine-sensitive strain of *Plasmodium berghei* NK65 and one hundred and fifteen (115) adult Swiss albino mice of both sexes and average weight 20 ± 2 g obtained from the University of Ilorin, Ilorin, Nigeria, Institute for Advanced Medical Research and Training (IAMRAT), College of Medicine, University of Ibadan, Nigeria and the Central Animal House of the University of Ibadan, Ibadan, Nigeria respectively.

### Ethical Clearance

Ethical clearance for all animal experiments (reported in the supplementary information) was obtained from the University Ethical Review Committee, University of Ilorin, Nigeria (Ethical clearance certificate number: UERC/ASN/2016/1087), according to the guidelines stipulated for animal care (Clark *et al*.^38^ and Garber *et al*.^39^).

### Synthesis

The unmodified mesoporous silica nanoparticles were synthesized according to a procedure previously reported^30^. Hexadecyltrimethylammonium bromide (CTAB), 1.9 g, 5.3 mmol was suspended in distilled water (480 mL) and raised the temperature to 50 °C to form a clear solution. Thereafter, NaOH (2 M) solution (Ca 4 mL) was added to the solution (pH = 12–13) with constant stirring and when the temperature reached 80 °C the tetraethyl orthosilicate (TEOS) (9.2 ml, 43.9 mmol) was dropped slowly and stirred for 2 h. The resulting product was filtered and washed severally with distilled water and methanol until the pH is 7.0 dried in vacuo overnight. The as-synthesized MCM-41 produced was calcined at 550 °C for 5 h to remove the surfactant template. Whereas, 3-phenylpropyl silane organo functionalized MCM-41 (pMCM-41) and their nanodrugs **1** and **2** were prepared according to literature with slight modifications.

### Characterization

A Fourier Transform Infrared (FT-IR) spectroscopy spectrophotometer recorded on FTIR-8700 spectrometer was used to record infrared spectra of the MSNs. The samples were grounded with KBr to form pellet and KBr background spectrum was used for determining the samples’ spectra over the range of 400~4000 cm^−1^. The interior pores properties like surface area, pore size and volume were determined with nitrogen physisorption measurements at −196 °C by using a Micromeritics Tristar 3000 system. MCM-41, pMCM-41 orgno-functionalized Silica material, and their QN encapsulated samples were degassed respectively at 120 °C for 6 h on a vacuum line. The pore-size distribution was measured from the desorption branch of the isotherm using BJH model followed by gaussian fitting specific and surface area of the samples were calculated by using the Brunauer–Emmett–Teller (BET) equation methods. Differential scanning calorimetric study was performed on NETZSCH DSC 200F3 Maia using the crimp-aluminum pan. The measurement was carried out from 20 to 200 °C at heating rate of 10 K/min under nitrogen gas flow for two cycles to check the structural phase transition peak of the samples’ molecular state. Thermogravimetric analyses were performed using a TG/DTA 6300 SII Seiko Instrument Inc, Japan at a heating rate of 10 °C/min under a nitrogen purge of 40 ml/min. Powder X-ray diffraction (PXRD) patterns of the silica and silica loaded drugs were recorded on a Smart Lab Rigaku X-ray diffractometer with Fe-filtered Co radiation (Rigaku Corporation, Japan). The measurement conditions were as follows: target, CuKa; filter, Ni; voltage, 30 kV; current, 15 mA; scanning range, 2–25°; scanning speed, 4 °C/min to measure the molecular state of the adsorbed drug. Transmission electron microscopy (TEM) structural images were obtained using a JEOL 1010 operated at 100 kV while JEOL-JSM 7600 F operating at an accelerating voltage of 5.0 kV was used to obtain field-emission Scanning Electron Microscope (SEM) micrographs/morphology of both unmodified and modified MSNs and their encapsulated materials. At first, the silica nanoparticles were dropped on the carbon-coated copper grid and then sputtered with thin film of platinum and gold to boost the silica conductivity before scanning. The amount of QN loaded and released from mesoporous silica were determined using UV-3600 UV-VIS spectrophometer Shimadzu.

## Electronic supplementary material

Supporting Information

## References

[1] World Health Organization (WHO) Global Malaria Programme, World Malaria Report (2015). http://apps.who.int/iris/bitstream/10665/200018/1/9789241565158_eng.pdf?ua=1d accessed February 20 (2017).

[2] Vasconcelos T, Sarmento B, Costa P (2007). Solid dispersions as strategy to improve oral bioavailability of poor water soluble drugs. Drug Discovery Today.

[3] Kwon, I. K., Jeong, S. H., Kang, E. & Park, K. Nanoparticle drug delivery Systems for Cancer therapy. *Cancer Nanotechnol*. 333–344 (2007).

[4] Ferris DP (2009). Light – operated mechanized nanoparticles. J. Am Chem. Soc..

[5] Snow RW, Guerra CA, Noor AN, Myint HY, Hay SI (2005). The Global Distribution of Clinical Episodes of *Plasmodium Falciparium* Malaria. Nature..

[6] Simon I. H. *et al*. Estimating the Global Clinical Byrden of *Plasmodium falciparum*. *Malaria, Plos Med* **7** (2007). 10.1371/journal.pmed.1000290 PMC2885984 20563310

[7] Fidock DA, Rosenthal PJ, Croft SL, Brun R, Nwaka S (2004). Antimalarial Drug discovery: Efficacy models for compound screening. Nature reviews.

[8] Adebayo JO, Santana AEG, Krettli AU (2012). Evaluation of the Antiplasmodial and Cytotoxicity potentials of husk fibre extracts from *Cocos nucifera*, a medicinal plant used in Nigeria to treat human malaria. Human and Experimental Toxicology3.

[9] Balogun EA, Akinloye OA, Lasisi AA, Adeyi OE (2012). Biochemical and histological changes associated with treatment of malaria and diabetes mellitus in mice with extracts of Marmodiaca charantia. Biokemstri.

[10] Rathee P, Dalal A, Kumar A, Ruhil M (2015). Nanotechnology A potential tool in malarial chemotherapy-review. International Journal of Multidisciplinary Research and Development.

[11] Santos-Magalhaes NS, Mosqueira VCF (2010). Nanotechnology applied to the treatment of malaria. Advanced drug delivery reviews.

[12] Vaidya, A. B. *et al*. Pyrazoleamide compounds are potent antimalarials that target Na+ homeostasis in intraerythrocytic Plasodium falciparum, *Nature communication*, DOI (2014). 10.1038/ncomms6521 PMC4263321 25422853

[13] Lasisi, A. A., Olayiwola, M. A., Balogun, S. A., Akinloye, O. A. & Ojo, D. A. Phytochemical composition, cytotoxicity and *in vitro* antiplasmodial activity of fractions from Alafia barteriolive (Hook F. Icon) -Apocynaceae. *Journal of Saudi Chemical Society* (2012).

[14] Amolegbe SA (2017). Some non toxic metal-based drugs for selected prevalent tropical pathogenic diseases. J. Biol Inorg Chem.

[15] Gabizon A, Papahadjopoulos D (1988). Liposomes formulations with prolonged circulation time in blood and enhanced uptake by tumors. P. Natl Acad Sci USA.

[16] Mathews OA, Shipway AN, Stoddart JF (1998). Dendrimers-branching out from curiosities into new technologies. Prog Polym Sci..

[17] Lee ES, Na K, Bae YH (2005). Doxorubicin loaded pH-sensitive polymeric micelles for reversal of resistant MCF-7 tumor. J. Control Release.

[18] Zhang H (2011). Synthesis of novel mesoporous silica nanoparticles for loading and release of ibuprofen. Journal of Controlled Release.

[19] Yang S (2006). On the origin of helical mesostructures. J. Am. Chem. Soc..

[20] Beck JS (1992). A new family of mesoporous molecular sieves prepared with liquid crystal templates. J. Am. Chem. Soc..

[21] Slowing I, Trewyn BG, Lin VS (2006). Effect of surface function of MCM-41 type mesoporous silica nanoparticles on the endocytosis by human cancer cells. J. Am. Chem. Soc..

[22] Francisco de J, Edwardo R-H (2000). Selective Functionalization of Mesoporous Silica. Adv.Mater.

[23] Urban P (2014). Use of poly (amidoamine) drug conjugates for the delivery of antimalarials to plasmodium. Journal of Controlled Release.

[24] Movellan J (2014). Amphiphilic dendritic derivatives as nanocarriers for the targeted deloivery of antimalarial drugs. Biomaterials.

[25] Nosten F, White NJ (2007). Artemisinin-based combination treatment of falciparum malarial. Am. J. Trop. Med Hyg..

[26] Haas SH, Bettoni CC, Oliveira LK, Guterres SIS, Costa TD (2009). Nanoencapsulation increases quinine antimalarial efficacy against *Plasmodium*. bergheiin viv., Int. J. Antimicrob. Agents.

[27] Culli PR (1997). Influence of pH gradients on the transbilayer transport of drugs, lipids, peptides and metal ions into large unilamellar vesicles, BBA-Rev. Biomembranes.

[28] Gabriels M, Plaizier-Vercammen J (2003). Physical and Chemical evaluation of liposomes, containing artesunate. J. Pharm. Biomed. Anal..

[29] Amolegbe SA (2016). Synthesis of mesoporous materials as nano-carriers for an antimalarial drug. J. Mater. Chem., B.

[30] Jambhrunkar S, Karmakar S, Popat A, Yu M, Yu C (2014). Mesoporous silica nanoparticles enhance the cytoxicity of curmin. RSC Advances.

[31] Anderson MT, Martin JE, Odinek JG, Newcorner PP (1998). Surfactant-Templated Silica Mesophases Formed inWater: Cosolvent Mixtures. Chem. Mater.

[32] Zhang Q, Ye Z, Wang S-T, Yin J (2014). Facile one-pot synthesis of PEGylated monodisperse mesoporous silica nanoparticles with controllable particles sizes. Chinese Chemical letters.

[33] He Q, Shi J, Chen F, Zhu M, Zhang L (2010). An anticancer drug delivery system based on surfactant-templated mesoporous silica nanoparticles. Biomaterials.

[34] Zhu Y, Fang Y, Borchardt L, Kaskel S (2011). PEGylated hollow mesoporous silica nanoparticles as potential drug delivery vehicles. Microporous and Mesoporous Materials.

[35] Zhang X (2014). Biofunctionalized polymer-lipid supported mesoporous silica nanopartciles for release of chemotherapeutics in multidrug resistant cancer cells. Biomaterials.

[36] Pan L, Liu J, He Q, Wang L, Shi J (2013). Overcoming multidrug resistance of cancer cells by direct intranuclear drug delivery using TAT-conjugated mesoporous silica nanoparticles. Biomaterials.

[37] Zhang Y (2012). Mesoporous Silica nanoparticles for Increasing the Oral Bioavailability and Permeation of Poorly Water SolubleDrugs. Mol. Pharmaceutic.

[38] Clark JD, Gebhart GF, Gouder JC, Keeling ME, Kohn DF (1997). The 1996 Guide for the care and use of laboratory animals. ILAR Journal.

[39] Garber, J. C. *et al*. Guide for the care and use ofLaboratory animals. Eight edition, T*he National Academic press*, 500 fifth street Washington DC 20055, (800) 624–6242 (2011).

