# Supplementary material for: Mesoporous silica nanocarriers encapsulated antimalarials with high therapeutic performance
Source: Sci Rep. 2018 Feb 15;8:3078. doi: 10.1038/s41598-018-21351-8 (PMC5814455; doi:10.1038/s41598-018-21351-8)
Supplement: Supplementary file 1 — Supporting Information [file 41598_2018_21351_MOESM1_ESM.docx]

Supplementary Information

**Mesoporous silica nanocarriers encapsulated antimalarials with high therapeutic performance**

Saliu AlaoAmolegbe, Yui Hirano, Joseph Oluwatope Adebayo, Olusegun George Ademowo, Elizabeth Abidemi Balogun, Joshua Ayoola Obaleye, Antoniana Ursine Krettli, Chengzhong Yu, Shinya Hayami

**Methods**

**QN loading in MCM-41 and pMCM-41**

Similar reported procedure^1^ was followed with slight change for loading the QN in the mesoporous silica nanoparticles. MCM-41 (40 mg) was soaked in 10 mL of ethanol solution of QN 10 mg (4:1 w/w) for 72h with constant mixing to allow full adsorption of the drug inside the silica, the mixture flask was covered with aluminium foil to protect the drug from light The drug loaded samples were separated from the solvent mixture by centrifugation for 30 minutes at 4000 rpm and washed with about 5 mL of ethanol to remove surface attached drugs. The residue i.e. adsorbed drug was dried in vacuum while the supernatant which contained small unabsorbed drug was determined after dilution using Beer-Lambert UV-Vis absorption spectrophotometer at 281.5 nm wavelength standard calibration curve. However, the actual amount of QN loaded in the MSNs was calculated by removing the weight of QN in the supernatant from the initial amount of QN used. The QN loaded MSNs were labeled as **1** and **2**.

**Equation formula for Drug Loading**

Drug loading capacity (**DLC %**) = Weight of QN in MSNs / weight of QN-silica composite 100%

Entrapment efficiency (**EE %**) = Weight of QN in MSNs / weight of initial QN loaded 100%

***In-vitro* quinine dissolution procedure**

The two prepared QN loaded silica equivalent to 2 mg of the QN drug were singly weighed and suspended in 1 ml of 0.5 % SLS buffer^1^. This suspension was then placed in dialysis bag (Sigma Aldrich) with 10 kDa molecular weight cutoff and was immersed into 9 ml of 0.5 % SLS at 37 ̊C with continuous stirring at pH 7.0. At predetermined time intervals, 1 mL of the samples were withdrawn and immediately replaced with an equal volume of dissolution medium to keep the volume constant. Pure QN release was studied along with silica-drug composite to compare the in vitro drug release profile by weighing 2 mg of pure QN and suspending it in 0.5 % SLS similar to that of MCM-41⊃QN These samples were then properly diluted and analyzed for QN content at 281.5 nm using UV-VIS spectrophotometer. pH effect on the dissolution was studied.

### ***In vivo* Antimalarial test**

### **Animal Handling**

The mice were acclimatized for one week prior to the commencement of experiment. The mice were housed in plastic cages. Environmental conditions were maintained at 25°C and a relative humidity of 50% with a 12 h light/dark cycle. The mice were fed normal mouse laboratory chow (Caps Feed, Ibadan, Nigeria), and given access to distilled water *ad libitum* throughout the study.

###

### **Antimalarial test**

The antimalarial activities of the antimalarial drug-loaded mesoporous silica nanoparticles were evaluated using the 4-day suppressive test described by Peters (1965).^2^ A donor mouse which had been previously infected with *Plasmodium berghei* NK-65 was obtained and tail blood sample was collected and used to prepare an inoculum size of 1x10^7^ of parasitized erythrocytes per 0.2 ml. This was used to inoculate each of the 115 mice intraperitoneally on day 0. The inoculated mice were then randomly distributed into twenty-three groups (of five mice each). Mice in group A were administered distilled water while mice in groups B and C were administered 5 mg/kg body weight artesunate and 15 mg/kg body weight quinine respectively. Mice in groups D1, D2, D3, D4 and D5 were administered 0.0625, 0.125, 0.25, 0.50 or 1.00 mg kg^-1^ body weight equivalent of artesunate (encapsulated in Calcined MCM-41) respectively. Mice in groups E1, E2, E3, E4 and E5 were administered 0.0625, 0.125, 0.25, 0.50 or 1.00 mg kg^-1^ body weight of equivalent quinine (encapsulated in Calcined MCM-41) respectively. Mice in groups F1, F2, F3, F4, and F5 were administered 0.0625, 0.125, 0.25, 0.50 or 1.00 mg kg^-1^ body weight equivalent of quinine (encapsulated in pMCM-41) while mice in groups G1, G2, G3, G4 and G5 were administered 0.0625, 0.125, 0.25, 0.50 or 1.00 mg kg^-1^ body weight equivalent of artesunate (encapsulated in aMCM-41).The nanodrugs/drugs were dissolved in distilled water before administration which was done orally 4 h post-inoculation on day 0. Treatment was repeated on days 1, 2 and 3 post-inoculations through the oral route.

Thin and thick smears were prepared from the tail of each mouse on days 4, 6 and 8 post-inoculations. The thin smears were fixed in methanol, air-dried, stained with Giemsa, and microscopically examined (Mag. x1000) by counting parasites in 1000 up to 6000 erythrocytes. Parasitaemia was and inhibition of parasite growth in the MSN encapsulated drug-treated groups and standard drug-treated groups in relation to the non-treated control group were calculated thus:

% Parasitaemia= Total number of infected RBC x 100

Total number of RBC

% Inhibition = Parasitaemia of non-treated group – Parasitaemia of treated group x 100

Parasitaemia of non-treated group

The dose-response relationship of the drug loaded nanoparticles was obtained by plotting the percentage inhibition against their respective doses. The ED_50_ values of the nanodrugs were then extrapolated from the curve. It was determined using Origin Software (Hilti, U.S.A). The mean survival time for five (5) mice in each group was determined arithmetically by finding the average of the survival time (days) of the mice post-inoculation over a period of 30 days.

**Notes**

Ethical Approval

Ethical clearance for all animal experiments was obtained from the University Ethical Review Committee, University of Ilorin, Nigeria (Ethical clearance certificate number: UERC/ASN/2016/1087), according to the guidelines stipulated for animal care (Clark *et al.*^3^ and Garber *et al.*^4^)

**MTT assay for *in vitro* cytotoxicity test**

The cytotoxicity test was done using a monkey kidney cell line (BGM), received from the Federal University of Minas Gerais, Belo Horizonte, Brazil. The test was performed as described by Shayne et al.^5^. Cells werecultured in 75 cm^2^ plates with RPMI 1640 medium supplemented with 10% fetal bovine serum (FBS) and gentamicin 40 mg/L, at 5% CO_2_ atmosphere and 37°C. The cells were trypsinized when the monolayer was confluent, washed with culture medium, distributed in a flat-bottomed 96-well plate (1×10^5^ cells/mL) and incubated at 37°C for 18 h to ensure cell adherence. The cells were properly diluted and incubated with 20 µL of the compounds at different concentrations (1-1,000 µg/mL) for 24 h in a 5% CO_2_ atmosphere at 37°C. The neutral red assay as described by Borenfreund and Borrero^6^ was used to evaluate cell viability by the accumulation of dye in the viable cell lysosome. 200 µL of neutral red solution (4mg/mL), was added to the plates and incubated for 3 h. The supernatant was carefully removed, followed by the addition of 200 µL of formaldehyde (0.5% v/v) and CaCl_2_ (1%) solution. After 5 min, the supernatant was removed, then 100 µL of analcohol- acetic acid (50:1%) solution was added to extract the dye. The absorbance was read at 540 nm on an ELISA reader (SpectraMax340PC384, Molecular Devices). Cell viability was expressed as the percentage of control absorbance obtained in untreated cells and the minimum lethal dose for 50% of the cells (MLD_50_) was determined.

**Antiplasmodial test**

The method of Trager and Jensen^7^ was used in culturing *P. falciparum* parasites in human red blood cells,with minor modifications. Briefly, parasites were cultured in Petri dishes (Corning, Santa Clara, CA, USA) containing RPMI culture medium [supplemented with 1% (v / v) albumax II (Gibco, USA)] with 5% hematocrit. Plates were incubated at 37°C, using the candle jar method. The culture medium was changed daily and parasitaemia monitored in Giemsa-stained smears. The parasites were synchronized with sorbitol solution as described by Lambros and Vandenberg^8^ to get predominantly ring forms, diluted and incubated in 96 well plates containing the extracts and the standard drug, or culture medium with 0.5% DMSO, used as a positive control of parasite growth.

The SYBR test was used as described by Smilkstein et al.^9^ with some modifications. Briefly, serial dilutions of nanodrugs were incubated at 37°C with the parasite suspensions (0.5% parasitaemia and 2% hematocrit) in"U" bottom 96-wells plates. After 48 h, the culture supernatant was removed and replaced by 100µL of lysis buffer solution [Tris (20 mM; pH 7.5), EDTA (5 mM), saponin (0.008%; wt/vol), and Triton X-100 (0.08%; v/v)] followed by addition of 0.2 μL/mL Sybr Safe (Sigma-Aldrich, Carlsbad, CA, USA). The plate content was then transferred to a flat bottom plate and incubated in the dark for 30 minutes. The reading was made in a fluorometer (Synergy H4 Hibrid Reader, BioteK) with excitation at 485 nm and emission at 535 nm.

**Supplementary Figure 1.** PXRD patterns of MCM-41, quinine (QN), **1** and**2**.

**Supplementary Figure 2.** FTIR spectra for **MCM-41** (red line), as-synthesized **MCM-41** (orange line) and **pMCM-41** (blue line).

**Supplementary Figure 3.**FTIR spectra for **1** (red line), **2** (blue line) and **QN** (orange line).

.

**Supplementary Figure 4.** DSC curves of(a) Quinine(**QN**), (b) **MCM-41**, (c) **pMCM-41**,(d) MCM-41 loaded QN (**1**) (e) pMCM-41⊃QN(**2**).

**Supplementary Figure 5.** TEM image of MCM-41hexagonal structure

**Supplementary Figure 6.**TGA of(a)QN,(b) MCM 41⊃QN(**1**),(c) pMCM-41⊃QN(**2**), (d) pMCM-41and (e) MCM-41.

**Supplementary Figure 7.** N_2_ adsorption/desorption curves of (a) **MCM-41**, (b) **1**, (c) pMCM-41 and (d) **2**.Adsorption isotherm curve is red line, desorption isotherm curve is blue line, respectively

References

1. Jambhrunkar, S., Karmakar, S., Popat, A., Yu, M.& Yu, C. Mesoporous silica nanoparticles enhance the cytoxicity of curmin, RSC . Advances, **4**, 709-712 (2014).
2. Peters, W. Chloroquine resistance Exp. Parasitology, **17**(1): 80-89 (1965).
3. Clark, J.D., Gebhart, G.F., Gouder, J.C., Keeling, M.E. &Kohn, D.F. The 1996 Guide for the care and use of laboratory animals. *ILAR Journal,***38**, 41-48 (1997).
4. Janet, C. G. *et al.* Guide for the care and use of Laboratory animals. **Eight edition**, *The National Academic press*,500 fifth street Washington DC 20055, (800) 624-6242. (2011).
5. Shayne C. Application of *in vitro* techniques in drug safety evaluation (2009).
6. Borenfreund, E.&Borrero, O. *in vitro* cytotoxicity assays, Potential alternatives to the Draize ocular allergy test Cell *BiolToxicol.***1**(1), 55-65 (1984).
7. Trager, W.&Jensen, J.B. Human malaria parasites in continuous culture, *Science*, **193**(4254): 673-675 (1976).
8. Lambros, C. & Vanderberg, J. Synchronization of *Plasmodium falciparum*erythrocytic stages in culture. *J. Parasitol***65**, 418-420 (1979).
9. Smilksten, M., Sriwilaijaroen, N., Kelly, J.X., Wilairat, P.&Riscoe, M. Simple and inexpensive fluorescence-based technique for high throughout antimalarial drug screening, *Antimicrob. Agents, Chemother***48,**1803-1806 (2004).
